# Impact of Doxycycline as Malaria Prophylaxis on Risk of Influenza-Like Illness among International Travelers

**DOI:** 10.4269/ajtmh.19-0648

**Published:** 2020-02-10

**Authors:** Kathryn Lago, Kalyani Telu, David Tribble, Anuradha Ganesan, Anjali Kunz, Charla Geist, Jamie Fraser, Indrani Mitra, Tahaniyat Lalani, Heather Yun, for the Infectious Disease Clinical Research Program TravMil Study Group

**Affiliations:** 1 Brooke Army Medical Center, Fort Sam Houston, Texas;; 2 Uniformed Services University of the Health Sciences, Bethesda, Maryland;; 3 Department of Preventive Medicine and Biostatistics, Infectious Disease Clinical Research Program, Uniformed Services University of the Health Sciences, Bethesda, Maryland;; 4 The Henry M. Jackson Foundation for the Advancement of Military Medicine, Inc., Bethesda, Maryland;; 5 Walter Reed National Military Medical Center, Bethesda, Maryland;; 6 Madigan Army Medical Center, Tacoma, Washington;; 7 Landstuhl Regional Medical Center, Landstuhl, Germany;; 8 Naval Medical Center, Portsmouth, Virginia

## Abstract

Travelers are often at risk for both influenza-like illness (ILI) and malaria. Doxycycline is active against pathogens causing ILI and is used for malaria prophylaxis. We evaluated the risk factors for ILI, and whether the choice of malaria prophylaxis was associated with ILI. TravMil is a prospective observational study enrolling subjects presenting to military travel clinics. Influenza-like illness was defined as subjective fever with either a sore throat or cough. Characteristics of trip and use of malaria prophylaxis were analyzed to determine association with development of ILI. Poisson regression models with robust error variance were used to estimate relative risk (RR) of ILI. A total of 3,227 trips were enrolled: 62.1% male, median age of 39 years (interquartile range [IQR] 27,59), median travel duration 19 days (IQR 12, 49); 32% traveled to Africa, 40% to Asia, and 27% to the Caribbean and Latin America. Military travel (46%) and vacation (40%) were most common reasons for travel. Among them, 20% took doxycycline, 50% other prophylaxis, and 30% took none; 8.7% developed ILI. Decreased RR of ILI was associated with doxycycline (RR 0.65 [0.43–0.99], *P* = 0.046) and military travel (RR 0.30 [0.21–0.43], *P* < 0.01). Increased risk of ILI was associated with female gender (RR 1.57 [1.24–1.98], *P* < 0.01), travel to Asia (RR 1.37 [1.08–1.75], *P* = 0.01), and cruises (RR 2.21 [1.73–2.83], *P* < 0.01). Use of doxycycline malaria prophylaxis is associated with a decreased risk of ILI. Possible reasons include anti-inflammatory or antimicrobial effects, or other unmeasured factors. With few strategies for decreasing ILI in travelers, these findings bear further investigation.

## INTRODUCTION

International travel for leisure, business, and/or military activities is increasingly common. With increased travel, there is increased risk of travel-related illness such as malaria, traveler’s diarrhea, and influenza-like illness (ILI). According to the 2011 GeoSentinel surveillance data, 23.3% of travelers reported a febrile illness. Of those febrile travelers, 29% were diagnosed with malaria and 10.9% were diagnosed with an ILI.^1^ Prevention of travel-related illness involves pretravel counseling, immunizations, and chemoprophylaxis. The mainstay of ILI prevention is hand hygiene and influenza vaccination,^2^ although the efficacy of these in preventing travel-related ILI is unknown.

Doxycycline is commonly used for malaria prophylaxis among international travelers. It is known to have activity against numerous respiratory pathogens, several pathogens associated with fever in the returning traveler, and has general anti-inflammatory effects but has not been evaluated to our knowledge for an association with development of ILI in travelers.^3^ In a cohort of travelers using a variety of malaria preventive strategies, we sought to evaluate whether the use of doxycycline as malaria prophylaxis was associated with a decreased risk of subjective ILI during international travel.

## MATERIALS AND METHODS

### Study design.

This study was a subset of the larger TravMil study: *Deployment and Travel Related Infectious Disease Risk Assessment, Outcomes, and Prevention Strategies Among Department of Defense (DoD) Beneficiaries*, approved by the Uniformed Services University of the Health Sciences Institutional Review Board (Bethesda, MD). TravMil is a prospective observational cohort of DoD beneficiaries traveling outside the continental United States for ≤ 6.5 months. Consenting adults were enrolled pretravel at six military travels (Walter Reed National Military Medical Center, Bethesda, MD; Brooke Army Medical Center, San Antonio, TX; Naval Medical Center, Portsmouth, VA; Madigan Army Medical Center, Tacoma, WA; Naval Medical Center, San Diego, CA; and Landstuhl Regional Medical Center, Landstuhl, Germany) and in the pre-deployment setting between January 2010 and August 2018. Eligible travelers were limited to DoD beneficiaries (e.g., active duty military personnel, military retirees, USPHS, employees of Department of State, and military-dependent family members). Individuals were required to be traveling outside of the continental United States, Puerto Rico, Guam, Samoa, the U.S. Virgin Islands, Western or Northern Europe, Canada, or New Zealand for at least part of their itinerary.

### Survey data.

Pretravel enrollees completed a pretravel survey recording their demographics, travel characteristics, and prescriptions for malaria prophylaxis. Travelers were also provided a diary to record episodes of fever, cough, and sore throat during travel. Subjective ILI was defined as a subjective fever with either a sore throat or cough. Episodes of subjective ILI were analyzed for each registered trip. For each registered trip which could include multiple geographic regions, the traveler was prescribed doxycycline, other, or no malaria prophylaxis for the duration of that trip.

### Statistical analysis.

Univariate and multivariate analysis were performed to determine which trip and traveler characteristics, including use of malaria prophylaxis, were associated with development of subjective ILI. For univariate and multivariate analysis, a Poisson regression with robust error variance was used to estimate the relative risk (RR) of our count data. A robust error variance allowed for a tighter CI. Variables with a *P*-value of ≤ 0.05 on univariate analysis underwent a Poisson regression for multivariate analysis to assess independent risk factors. These criteria allowed for exclusion of highly correlated covariate and mutually exclusive variables. Results of multivariate analysis were reported with RR with 95% CIs. Statistical significance was defined as a *P*-value of ≤ 0.05. Statistical analysis was performed with SPSS software (IBM SPSS Statistics Version 22, Chicago, IL).

## RESULTS

### Baseline characteristics.

A total of 3,227 trips with 2,803 travelers were analyzed (Table 1). The majority of travelers were male (62.1%) with a median age of 39 years (IQR 27, 59) and with a median duration of travel of 19 days (IQR 12, 49). Caucasian (70.7%) was the most commonly reported race. Twenty-five percent of travelers were ≥ 60 years old and 39% who developed subjective ILI were ≥ 60 years old. For malaria prophylaxis, 20% were prescribed doxycycline, 50% prescribed another form of malaria prophylaxis, and 30% were prescribed no malaria prophylaxis. Of the other malaria prophylaxis group, 89% were atovaquone–proguanil. Travelers’ itineraries could include more than one region of travel. Forty percent traveled to Asia, 32% traveled to Africa, 28% traveled to the Caribbean, Mexico, Central America, and South America. The cumulative incidence of subjective ILI was 9% with an incidence rate of 26.5 per 1,000 travel days.

**Table 1:** Traveler demographics, malaria prophylaxis prescribed, destinations, and incidence of ILI

Characteristics	All (*N* = 3,227)	Non-deployment travel (*N* = 2,154)	Deployment (*N* = 1,073)
Gender, male	2,004 (62%)	1,106 (52%)	898 (84%)
Median age (years)	39 (IQR 27,59)	51 (IQR 33,65)	28 (IQR 23,35)
Median duration of travel (days)	19 (IQR 12,49)	16 (IQR 11,27)	34 (IQR 16, 84)
Race			
African American	345 (11%)	244 (11%)	101 (9%)
Caucasian	2,252 (71%)	1,508 (70%)	744 (69%)
Military status			
Active duty	1,658 (51%)	585 (27%)	1,073 (100%)
Dependent	1,454 (45%)	1,454 (68%)	-
Malaria prophylaxis prescribed			
Doxycycline	644 (20%)	174 (8%)	470 (44%)
Other*	1,623 (50%)	1,235 (57%)	388 (36%)
None	960 (30%)	745 (35%)	215 (20%)
Destinations			
Africa	1,033 (32%)	661 (31%)	372 (35%)
Asia	1,299 (40%)	752 (35%)	547 (51%)
Caribbean, Mexico, and Central America	704 (22%)	583 (27%)	121 (11%)
South America	356 (11%)	332 (15%)	24 (2%)
Multiple regions	1,013 (31%)	519 (24%)	494 (46%)
ILI cumulative incidence	281 (9%)	265 (12%)	16 (2%)

ILI = influenza-like illness.

* 89% atovaquone–proguanil.

More than one purpose of travel and multiple types of travel accommodations could be reported (Table 2). The most common purposes of travel were military (46%) and vacation (40%). Only 3% of travelers who traveled for military purposes were non-active duty (representing government employees/contractors). The most common travel accommodation was hotel (71%). Eleven percent of travelers went on a cruise. Seventeen percent of those cruise ship travelers were traveling for military purpose.

**Table 2:** Purpose of travel, travel accommodations, and type of travel location

Characteristic	All (*N* = 3,227)	Non-deployment travel (*N* = 2,154)	Deployment (*N* = 1,073)
Purpose of travel			
Military	1,468 (46%)	395 (18%)	1,073 (100%)
Vacation	1,282 (40%)	1,282 (40%)	–
Visiting friends and relatives	386 (12%)	386 (12%)	–
Medical	148 (5%)	148 (5%)	–
Travel accommodations			
Hotel	2,295 (71%)	1,814 (84%)	481 (45%)
Cruise	354 (11%)	314 (15%)	40 (4%)
Military accommodations	198 (6%)	198 (6%)	–
Private accommodations	398 (12%)	398 (12%)	–
Type of location			
Rural	1,752 (54%)	1,036 (48%)	716 (67%)

### Statistical analysis.

On univariate analysis of travel characteristics (Table 3), female travelers, travelers in Asia, South America, and longer duration of travel were associated with increased risk of developing subjective ILI. Vacation, visiting friends and relatives, and cruise ship travel were associated with increased risk of developing subjective ILI. On univariate analysis, use of doxycycline for malaria prophylaxis (compared with the use of other or no prophylaxis, combined to optimize sample size), travel to Africa, and military purpose of travel were associated with decreased risk of subjective ILI. In addition to doxycycline, gender, military purpose of travel, travel to Asia, cruise ship travel, and duration of travel were chosen as variables for multivariate analysis. Gender and cruise ship travel were selected because they had a strong effect on the univariate analysis and there have been several published studies on these variables and ILI rates. Military purpose of travel was chosen as this is one of the key populations in the TravMil group and to ensure that doxycycline use and military travel were both considered in the model simultaneously. Travel to Asia was chosen for multivariate analysis as it represents the largest area of travel in our study, and was also significantly associated with ILI on univariate analysis. On multivariate analysis (Table 4), use of doxycycline (0.65 [0.43–0.99], *P* = 0.046) and military purpose of travel (0.3 [0.21–0.43], *P* < 0.01) remained significantly associated with decreased risk. On multivariate analysis, female gender (1.57 [1.24–1.98], *P* < 0.01), median duration of travel in days (1.0 [1.0–1.01], *P* < 0.01), travel to Asia (1.37 [1.08–1.75], *P* = 0.01), and cruise ship travel (2.21 [1.73–2.83], *P* < 0.01) were associated with increased risk.

**Table 3:** Univariate analysis of characteristics and their RR of ILI

Characteristic	ILI	No ILI	Univariate RR (95% Cl)	Univariate *P*-value
Malaria prophylaxis				
Doxycycline	29 (5%)	615 (95%)	0.46 (0.32–0.67)	< 0.01
Other or none	252 (10%)	2,331 (90%)	Ref	
Other	144 (9%)	1,479 (91%)	0.78 (0.62–0.99)	
None	108 (11%)	852 (89%)	Ref	
Gender				
Male	121 (6%)	1,885 (94%)	Ref	
Female	160 (12%)	1,061 (88%)	2.16 (1.73–2.72)	< 0.01
Age (years, IQR)	54 (31,66)	38 (27,57)	–	< 0.01
Median duration of travel (days, IQR)	21 (14,36)	19 (12,42)	–	0.03
Destinations				
Africa	75 (7%)	958 (93%)	0.77 (0.60–0.99)	0.05
Asia	133 (10%)	1,166 (90%)	1.26 (1.00–1.58)	0.04
South America	46 (14%)	286 (86%)	1.70 (1.27–2.29)	0.04
Mexico/Caribbean/Central America	53 (9%)	557 (91%)	0.99 (0.75–1.32)	0.99
Multiple regions	93 (9%)	920 (91%)	1.08 (0.85–1.37)	0.52
Purpose of travel				
Military	53 (4%)	1,415 (96%)	0.28 (0.20–0.37)	< 0.01
Vacation	176 (22%)	1,106 (78%)	2.54 (2.02–3.20)	< 0.01
Visiting friends and relative	46 (12%)	340 (88%)	1.44 (1.06–1.94)	0.02
Medical	10 (7%)	138 (3%)	0.76 (0.42–1.41)	0.39
Travel accommodations				
Hotel	180 (10%)	1,666 (90%)	1.33 (1.06–1.68)	0.02
Cruise*	73 (21%)	281 (79%)	2.84 (2.23–3.63)	< 0.01
Military	20 (10%)	178 (90%)	1.17 (0.76–1.80)	0.47

ILI = influenza-like illness; RR = relative risk.

* Including deployment on USS comfort.

**Table 4:** Multivariate analysis of characteristics and their RR of influenza-like illness

Characteristic	Multivariate RR (95% Cl)	Multivariate *P*-value
Doxycycline	0.65 (0.43–0.99)	0.046
Female	1.57 (1.24–1.98)	< 0.01
Median duration of travel (days)	1.01 (1.0–1.01)	< 0.01
Asia	1.37 (1.08–1.75)	0.01
Military travel	0.30 (0.21–0.43)	< 0.01
Cruise	2.21 (1.73–2.83)	< 0.01

RR = relative risk.

### Military subgroup analysis.

Because 82% of the doxycycline group were traveling for a military purpose, a subgroup analysis was performed (Table 5). A multivariate Poisson (performed with the same variables as aforementioned, with the exception of military purpose of travel because this was the subset being analyzed, and with the addition of Africa because of this being the second most common region of deployment and also significant on univariate analysis) showed that doxycycline continued to have a statistically significant decreased RR of subjective ILI (0.42 [0.19–0.91], *P* = 0.03). Female gender (2.43 [1.39–4.25], *P* < 0.01) and cruise ship travel (4.6 [2.28–9.26], *P* < 0.01) continued to have a statistically significant increased RR of subjective ILI.

**Table 5:** Multivariate analysis of characteristics and their RR of influenza-like illness in military subgroup

Characteristic (*N* = 1,468)	Multivariate RR (95% Cl)	*P*-value
Doxycycline	0.42 (0.19–0.91)	0.03
Female	2.43 (1.39–4.25)	< 0.01
Median duration of travel (days)	1.0 (0.99–1.01)	0.10
Asia	1.2 (0.57–2.5)	0.65
Africa	1.2 (0.54–2.71)	0.63
Cruise	4.6 (2.28–9.26)	< 0.01

RR = relative risk.

## DISCUSSION

Our evaluation of the use of doxycycline as malaria prophylaxis and risk of subjective ILI among international travelers is to our knowledge the only such report in the medical literature and demonstrates a negative association between doxycycline used as malaria prophylaxis and incidence of subjective ILI in international travelers. Given the widespread prevalence of ILI in international travelers, as well as the limitations and unclear efficacy of the currently recommended interventions for prevention in this context, these findings warrant consideration. Although these observational data cannot be used to determine causality, both antimicrobial and anti-inflammatory effects of the drug suggest the possibility of a biologically plausible effect. Because there are limited data on chemoprophylaxis strategies for ILI and the effect of doxycycline on the microbiology of ILI, we explored literature of uses of doxycycline for prevention of lower respiratory disease.

Empiric uses of doxycycline for prophylaxis or treatment of respiratory infection have been recommended outside of travel medicine. The use of doxycycline for prophylaxis in patients with chronic obstructive pulmonary disease (COPD), to decrease the frequency of exacerbations and improve quality of life, has been well described.^4^ In 2007, the British Thoracic Society published guidelines for treatment on ILI during an influenza pandemic. They recommended empiric use of doxycycline or amoxicillin–clavulanate for bacterial pneumonia in COPD or severe preexisting illness.^5^ During the 2009 H1N1 pandemic, the United Kingdom Department of Health re-emphasized the use of empiric antibiotics for high-risk patients presenting with ILI.^6^ Further analysis of patients presenting with post-influenza bacterial pneumonia to outpatient care centers throughout the United Kingdom from 2007 to 2010 showed that 96% of bacterial respiratory isolates were susceptible to doxycycline.^7^ According to the Alexander Project, a large multinational surveillance study of respiratory pathogens, 71.3% of *Streptococcus pneumoniae*, and 95.8% of *Moraxella catarrhalis* are susceptible to doxycycline.^8^ The Infectious Disease Society of America recommends use of doxycycline as an alternate monotherapy for community-acquired pneumonia for healthy individuals who are not at risk of having drug-resistant *Streptococcus pneumoniae* infections.^9^ Doxycycline has been shown to have a wide empiric coverage of bacterial respiratory pathogens, which could explain the decreased RR of developing subjective ILI as seen in our study, to the extent that bacterial pathogens contribute to ILI, which is unclear.

Specific bacterial causes of respiratory infection in international travelers are less well defined, and it is challenging to know what proportion of these may be covered by doxycycline. Previous studies have reported influenza rates among returning travelers to be from 6 to 30%^10,11^ and streptococcal pharyngitis rates around 6%.^10^ However, some studies with ILI in the returning traveler only look for viral pathogens as the etiology of their respiratory symptoms.^11,12^ GeoSentinel, which only captures those travelers who are ill enough after travel to seek medical care and records specific diagnoses as they have been recorded in the course of usual clinical care, reports only 35 cases of *Legionella*, 30 cases of pertussis, and two cases of diphtheria out of its 600 travelers reporting respiratory illnesses.^1^ With these reports of bacterial pathogens as the cause of respiratory illness in returning travelers, it is microbiologically plausible that doxycycline may be covering some of these pathogens.

The military is a unique population of travelers who may be at higher risk for acquiring respiratory pathogens as they stay in crowded accommodations, interact with local populations, and may have limited sanitation. In a retrospective study, deployers to Operation Enduring Freedom had pre- and post-deployment sera taken to look for rates of respiratory pathogen seroconversion. One of seven deploying personnel seroconverted to a respiratory pathogen. Of note, there was a 10% seroconversion rate to *Chlamydia pneumoniae,* 4.3% conversion rate to *Bordetella pertussis,* and 3.7% conversion rate to *Mycoplasma pneumoniae.* The seroconversion rate of *B. pertussis* is two to four times higher than the adult U.S. population.^13^ In addition, GeoSentinel showed that respiratory bacterial infections such as streptococcal pharyngitis, sinusitis, and otitis media were significantly greater in younger persons with median ages in the 20–30s, which is similar to our study population.^10^ Therefore, our study population could be experiencing a slightly higher rate of bacterial pathogens than the normal travel population. Of importance, GeoSentinel clinics use multiple diagnostic methods to determine etiologic agents of disease, and our study lacks pathogen identification. In addition, GeoSentinel clinics capture travelers presenting to their clinic at the time of their acute illness. However, most travelers with ILI will have their illness resolve before being evaluated at a travel clinic, as was the case in our study. Therefore, it is difficult to truly extrapolate the GeoSentinel population to in our study. Furthermore, it is challenging to determine if the antimicrobial properties are contributing to the decreased RR of subjective ILI seen in our study. Importantly, doxycycline has been used in medicine solely for its anti-inflammatory properties.

Tetracyclines have a regulatory influence on the immune system and inflammatory pathways.^14,15^ Doxycycline has been shown to inhibit activation of macrophages which in turn inhibits tumor necrosis factor (TNF)-α, interleukin (IL) 1b, IL-6, and other pro-inflammatory proteins. Tetracyclines also inhibit lipopolysaccharide-induced nitric oxide production. Nitric oxide production has been implicated in the pathophysiology of sepsis and rheumatoid arthritis.^14^ Doxycycline is currently being studied for its anti-inflammatory properties to treat allergen-induced inflammation, osteoarthritis, and corneal inflammation. Doxycycline also has anti-oxidant properties, thereby reducing tissue destruction.^16^ Therefore, the decreased RR of subjective ILI seen in our study could be related to the anti-inflammatory properties associated with doxycycline.

Several additional factors described in this study as being associated with the risk of ILI are consistent with previously published data. Associations with gender and the development of ILI, as well as the causes of ILI have been previously reported.^10,17,18^ A study of military travelers to Iraq and Afghanistan showed an increased RR of developing ILI in females.^17^ Similarly, GeoSentinel data have shown a statistically significant decreased odds ratio (OR) of developing upper respiratory infections in males. However, there was an increased OR of developing lower respiratory tract infection and pneumonia in males.^10^

Cruise ship travel has also been described for being associated with increased risk of acquiring an ILI among travelers.^10,19^ Pavli et al. described different respiratory pathogens acquired by cruise ship passengers and crew in a 3-year prospective study. The ILI attack rate was 0.6% among passengers and 1.3% among crew members. There were no reported cases of influenza A or B. However, rapid *Streptococcus* testing was positive in 4.5% of passengers and 30.9% of crew members.^19^

Our study had multiple limitations. First, our study collected observational data with self-reported ILI and subjective fever. Collecting subjective self-reported data introduces recall bias, and lack of pathogen identification makes it difficult to determine if the travelers truly had an ILI. In addition to lack of pathogen identification, it is not possible to know whether the decreased RR of subjective ILI with doxycycline could be due to its antimicrobial properties. Giving travelers a thermometer and real-time methods of collecting sputum and/or blood samples for polymerase chain reaction (PCR) could have allowed for more robust data to determine the association of reduced risk of ILI and doxycycline. In addition, because this is observational data, there may be potential unmeasured confounders. Some of those confounders include the possibility of unrecorded chronic diseases in nonmilitary members such as underlying cardiac or respiratory illnesses, smoking history, household contacts with respiratory illnesses in the week before travel, the effects of living or working in congregate settings, and the season of infection. An additional limitation to this study is that pneumococcal vaccination status and antibiotic use before or during travel were not analyzed.

Influenza season is dependent on geographic location. In our study, median travel duration was 19 days. However, on military deployment trips, the median duration was 34 days. In addition, subjects frequently traveled to multiple locations, 31%, and therefore, portions of their travel could be in different regions of influenza exposure. Because so many trips involved changes in season (including changes in seasonality based on geography), we did not explore seasons of travel and their effect on subjective ILI.

Influenza immunization and hand hygiene are recommended by the CDC to prevent ILI in international travelers.^2^ Active military personnel are required to receive annual influenza vaccination. Therefore, decreased rates of subjective ILI among military members may be secondary to immunization status. Previous TravMil studies^20^ have shown that there was no statistically significant effect on ILI with increasing number of influenza vaccines over a 5-year period; thus, this variable was not re-explored. This finding is in contrast to previously published data by Qureshi et al., which showed the influenza attack rate was lower among pilgrims per Haji who had influenza vaccine (attack rate of 36% versus 62%, respectively). However, in the Qureshi et al.^21^ study group, only 54% of subjects had pretravel vaccines.

One of the greatest strengths of our study include its large sample size with 3,227 trips recorded and including various types of malaria prophylaxis taken, which allowed for statistical analysis to compare the effects of doxycycline for prophylaxis versus other or no prophylaxis on subjective ILI incidence. Furthermore, our military subgroup analysis showed that use of doxycycline was still associated with a statistically significant decrease RR of subjective ILI, further strengthening our findings and decreasing the likelihood that the decrease in subjective ILI risk seen with doxycycline use may be attributed to some other military-specific aspect of travel.

## CONCLUSION

The cumulative incidence of subjective ILI in international travelers who were taking doxycycline for malaria prophylaxis was decreased compared with those taking other or no prophylaxis. This effect may be secondary to the multiple properties of doxycycline including, but not limited to: antibiotic, antiviral, anti-inflammatory, and anti-oxidant effects. The mainstay of ILI management in a traveler is prevention with good hand hygiene and influenza vaccine.^2^ With few other strategies known to decrease ILI, further investigation on the correlation between doxycycline use and incidence of ILI is warranted.
